# Preparation of Liposomal Formulations for Ocular Delivery of Thymoquinone: In Vitro Evaluation in HCEC-2 e HConEC Cells

**DOI:** 10.3390/pharmaceutics13122093

**Published:** 2021-12-05

**Authors:** Elisa Landucci, Francesca Bonomolo, Chiara De Stefani, Costanza Mazzantini, Domenico Edoardo Pellegrini-Giampietro, Anna Rita Bilia, Maria Camilla Bergonzi

**Affiliations:** 1 Department of Health Sciences, Section of Clinical Pharmacology and Oncology, University of Florence, Viale Pieraccini 6, 50139 Florence, Italy; elisa.landucci@unifi.it (E.L.); costanza.mazzantini@unifi.it (C.M.); domenico.pellegrini@unifi.it (D.E.P.-G.); 2 Department of Chemistry, University of Florence, Via Ugo Schiff 6, Sesto Fiorentino, 50019 Florence, Italy; francesca.bonomolo@stud.unifi.it (F.B.); chiara.destefani@stud.unifi.it (C.D.S.); ar.bilia@unifi.it (A.R.B.)

**Keywords:** thymoquinone, liposome, hyaluronic acid, corneal cells, conjunctival cells, cytotoxicity, uptake

## Abstract

Thymoquinone (TQ) is the main constituent of *Nigella sativa* L. essential oil. In vitro studies have shown its protective effect against H_2_O_2_-induced oxidative stress in human retinal pigment epithelium cells, and in vivo experiments have demonstrated its effect in decreasing corneal neovascularization and reducing the inflammation in an experimental dry eye model in mice. Its therapeutic use is limited by poor bioavailability, low solubility, and scarce permeability. In this study, two liposomal formulations have been developed, both of which consist of phosphatidylcholine and Plurol Oleique, a liquid lipid, and one of which is coated with 0.1% *w*/*v* hyaluronic acid (HA) to increase both TQ solubility and its ocular therapeutic potential. Each formulation has a size <200 nm and an EE% around 70%, determined by scattering techniques and the HPLC-DAD analytical method, respectively, and they result in a 2-fold increase in TQ solubility. HA-coated liposomes are stable over 2 months at +4 °C, and coated and uncoated liposomes present a gradual and prolonged release of TQ. Two cell lines, human corneal epithelial cells (HCEC-2) and human conjunctival epithelial cells (HConEC) were used to investigate the safety of the liposomal formulations. Uptake studies were also performed using fluorescent liposomes. Both liposomes and, in particular, HA-coated liposomes reduce the TQ toxicity observed at high doses in both HCEC-2 and HConEC cells, and both formulations increase the absorption at the cellular level and especially at the nucleus level, with a more pronounced effect for HA-coated liposomes.

## 1. Introduction

Thymoquinone (TQ), the main constituent of *Nigella sativa* L. seed (black seed) essential oil, has various pharmacological properties useful for the treatment of a wide range of diseases including chronic non-infectious diseases (neurological disorders, diabetes mellitus, hypertension, dyslipidemia, inflammatory disorders, cancer, etc.) and infectious diseases [1,2,3,4,5].

In particular, the protective effect of TQ against H_2_O_2_-induced oxidative stress in human retinal pigment epithelium cells has been reported [6]. TQ has shown anti-inflammatory properties in an experimental dry eye model, and it has decreased corneal neovascularization in a dose-dependent manner after topical administration in a rat model [7]. Furthermore, TQ acts on the immune system, modulating the levels of pro- and anti-inflammatory mediators that are involved in corneal neovascularization, and it is as effective as topical triamcinolone when topically applied to the eye at 0.4% [8].

The administration of TQ on ovalbumin (OVA)-induced allergic conjunctivitis in BALB/c mice significantly reduced the ocular symptoms of the allergic conjunctivitis, attenuating the recruitment of eosinophils, as well as the levels of IgE, histamine, and cytokines [9].

Despite its many activities [7], the clinical application of TQ is limited by poor bioavailability, low solubility, and scarce permeability. The in vitro studies have also shown toxicity of TQ depending both on the cell model and the dose [10]. The use of nanoformulations can represent a good tool for overcoming these limits [11,12]. The purpose of this study is to develop liposomal formulations of TQ, to improve its biopharmaceutical performance, and to enhance the delivery and the activity at the eye level. Liposomes, due to their unique structure, are extremely beneficial drug carriers as they can entrap both the hydrophilic and hydrophobic drugs [13]. Phospholipids are the major components of most liposomes. Extensive testing of these naturally occurring compounds has revealed them to be remarkably safe for pharmaceutical use. Liposomes represent the most effective ocular drug delivery carriers with sufficient flexibility to allow synthesis in various sizes, and they can also be easily administered in liquid dosage forms, such as eye drops, gels and ointments, for topical delivery [13]. Liposomes enhance corneal permeability due to their ability to come in close contact with cornea and conjunctiva, increasing the extent of corneal uptake and prolonging corneal contact time [13].

Moreover, liposomal formulations can also improve the stability and reduce the toxic side effects associated with the drug [14]. In addition, liposomes protect drug molecules from the metabolic enzymes at the tear/corneal epithelium interface [15]. Antiviral drugs (acyclovir, ganciclovir, idoxuridine), antibacterial drugs (tetracycline, gentamicin, tobramycin, ciprofloxacin, chloramphenicol), antifungal agents (amphotericin B, fluconazole), anti-inflammatory and immunomodulatory agents (diclofenac, cyclosporin, tacrolimus, 5-fluorouracil), and antiglaucoma agents (pilocarpine, latanoprost, acetazolamide) have been formulated in liposomal forms to enhance ocular bioavailability and activity [13,16].

TQ improved the retinal damage and the inflammatory reactions in an induced glaucoma model in rabbits when formulated in liposomes also containing latanoprost. This formulation conferred a sustained drug release and decreased the intraocular pressure [17]. Recently, vitamin E-loaded liposomes have been investigated for dry eye treatment. Liposomes were coated with 0.2% sodium hyaluronate, mimicking the aqueous phase of the mucin of the lacrimal film and prolonging contact time with the eye surface [18].

In this research, we combined the advantages of the liposomes for ocular delivery with a hyaluronic acid (HA) coating. HA is an endogenous negatively charged natural polysaccharide of d-glucuronic acid and *N*-acetyl-d-glucosamine disaccharide units, which is highly biocompatible, biodegradable, and non-toxic. In addition, it is a potential targeting moiety to cluster of differentiation 44 (CD44)-expressing cells, to the receptor for hyaluronan-mediated motility (RHAMM), and to Intercellular Adhesion Molecule 1 (ICAM-1). CD44 receptors are present in the ophthalmic cells, particularly in human conjunctiva and cornea epithelial cells, and in the retinal pigment epithelium [19,20].

Consequently, two liposomal formulations have been developed, both consisting of phosphatidylcholine and Plurol Oleique, a liquid lipid. One liposome was coated with 0.1% *w*/*v* HA to increase TQ solubility and availability at the ocular level.

The physical characterization of the formulations was carried out using scattering techniques (DLS and ELS), and transmission electron microscopy was used for the morphological analysis. Encapsulation efficiency was determined using the HPLC-DAD analytical method. The formulations were also submitted to stability studies over 2 months, keeping the samples at +4 °C, and to in vitro drug release studies at 37 °C. Two cell lines, human corneal epithelial cells (HCEC-2) and human conjunctival epithelial cells (HConEC), were used to verify the safety profile of the liposomal formulations. The same cells were used for the uptake studies, using fluorescent liposomes containing FITC as probes.

## 2. Materials and Methods

### 2.1. Materials

Acetonitrile HPLC grade, Cholesterol (CH), Fluorescein isothiocyanate (FITC, purity > 90%, HPLC), mucin from porcine stomach type II, TQ, and Tween 80, were purchased from Sigma-Aldrich (Milan, Italy). Capryol 90, Lauroglycol 90, Maisine CC, Plurol Oleique CC 497, and Transcutol P were supplied by Gattefossè sas (Saint-Priest, France). Phosphotungstic acid was purchased from Electron Microscopy Science (Hatfield, MA, USA). Sodium hyaluronate was obtained from Altergon Italia s.r.l. (Avellino, Italy). Egg phosphatidylcholine, Phospholipon 90 G was purchased by Phospholipid Gmbh (Cologne, Germany). Sodium hyaluronate (M.W. 1000 KDa, HA) was obtained from Altergon, Avellino, Italy. The water used was from the Milli-Qplus system from Millipore (Milford, CT, USA). The dialysis kit was from Spectrum Laboratories, Inc. (Breda, The Netherlands). The PAMPA filter plate (pore size 0.45 μm) was purchased from Millipore Corporation, Tullagreen, Carrigtwohill, County Cork, Ireland. Human corneal epithelial cells (HCE-2) were purchased from ATCC company (American Type Culture Collection, Manassas, VA, USA). Human conjunctival epithelial cells (HConEC) and corneal epithelium cell medium were provided by Innoprot (Derio, Bizkaia, Spain). Keratinocyte serum-free medium, bovine pituitary extract (BPE), and epidermal growth factor (EGF) were purchased from Gibco-BRL (San Giuliano Mil-anese, MI, Italy). Hydrocortisone, insulin, fibronectin, bovine collagen type I and bovine serum albumin (BSA) penicillin/ streptomycin (P/S), bovine fetal serum and poly-l-lysine, benzalkonium chloride (BAK), and 3-(4,5-dimethylthiazol-2-yl)-25-diphenyltetrazolium bromide (MTT) were obtained from Sigma (St Louis, MO, USA). The Cytotoxicity detection kit (lactate dehydrogenase, LDH) was obtained from Roche Diagnostics (Basel, Switzerland).

### 2.2. Chromatography Conditions and Instruments

#### HPLC Method for TQ and FITC

The HPLC system consisted of a 1100 High Performance Liquid Chromatograph (HPLC) equipped with a Diode Array Detector (DAD) from Agilent Technologies Italia Spa (Rome, Italy). The analytical column was a Kinetex C18 (150 × 4.6 mm, 5 μm Agilent Technology, Insert: Santa Clara, CA, USA).

The compounds were detected at a wavelength of 254 nm with an eluent flow rate of 0.8 mL/min, with (A) acetonitrile and (B) water pH 3.2 (by formic acid) as mobile phases, and with a gradient analytical method as described by [11]. The calibration curve, with a coefficient of determination R^2^ of 0.9993, was prepared using a standard solution of TQ in methanol (0.5 mg/mL) and successive dilutions of 5, 10, 50, 100, and 500-fold.

FITC analysis were performed using the same apparatus and the same column reported for TQ as well as the same method described by [21]. The calibration curve, with a coefficient of determination R^2^ of 0.9993, was prepared using a standard solution of FITC in methanol HPLC grade (1.0 mg/mL) and successive dilutions of 10, 50, 100, 200, and 500-fold.

### 2.3. Preparation of Liposomal Formulations

Blank liposomes (LP), TQ-loaded liposomes (LP-TQ), HA-coated liposomes (LP-TQ-HA), Fluorescein isothiocyanate (FITC)-loaded liposomes (LP-FITC), and HA-coated FITC-loaded liposomes (LP-FITC-HA) were prepared according to the thin layer evaporation method by [22]. For LP, 600 mg of egg phosphatidylcholine (PC) and 100 mg of Plurol Oleique were dissolved in dichloromethane. The organic solvent was evaporated under vacuum, and the dry lipid film was hydrated by adding 10 mL of deionized water. The aqueous dispersion was shaken for 30 min with a mechanical stirrer in a water bath at a temperature of 50 °C. To obtain unilamellar vesicles from multilamellar vesicles, an ultrasonic probe was used for 2 min and 30 s (with pulsed duty cycles of $\frac{1}{2}$ s on and $\frac{1}{2}$ s off, amplitude 48%); during the sonication, the sample was placed in an ice bath to prevent lipid degradation [23]. Finally, a centrifugation of 1 min at 1205× *g* was achieved to remove metallic particles potentially released by the ultrasonic probe inside the liposomal dispersion.

LP-TQ and LP-TQ-HA were prepared using the same method described above, adding 10 mg of TQ (1 mg/mL, corresponding to 1% of the weight of the lipid phase), together with PC, Plurol Oleique, and 1–2 mL of dichloromethane to completely dissolve the TQ. For LP-TQ-HA, the coating was achieved using the drop-wise method described by [24], adding 2 mL of 0.1% *w*/*v* of HA in deionized water to 2 mL of LP-TQ dispersion. The obtained dispersion was placed under magnetic stirring for 1 h, and then an ultrasonic probe was used for 2 min and 30 s (with pulsed duty cycles $\frac{1}{2}$ s on and $\frac{1}{2}$ s off, amplitude 48%). Finally, the sample was centrifugated for 1 min at 1205× *g*. The obtained formulation showed a final concentration of 0.5 mg/mL of TQ, as evaluated by HPLC-DAD analysis.

LP-FITC and LP-FITC-HA were prepared using the same methods described above, adding 10 mg of the FITC, corresponding to 1% of the weight of the lipid component.

### 2.4. Characterization of Liposomes

#### 2.4.1. Physical Characterization

Liposomes’ hydrodynamic diameter, polydispersity index (PdI), and zeta-potential were determined by Dinamic Light Scattering (DLS) and Electrophoretic Light Scattering (ELS), using a Zsizer Nano series ZS90 (Malvern Instruments, Malvern, UK). All samples were diluted 10-fold with deionized water, and an average of three measurements at stationary level was taken. A Haake temperature controller kept the temperature constant at 25 °C.

#### 2.4.2. Morphological Characterization

Morphological characterization was achieved by using the transmission electron microscopy (TEM) CM12 from Philips with an accelerating voltage of 80 kV. The aqueous dispersion was diluted 10-fold in deionized water, and 10 μL was applied to a 150-mesh carbon film-covered copper grid. To obtain a thin film, excess sample was eliminated from the grid with a filter paper. After that, 5 μL of phosphotungstic acid solution (1% *w*/*v* in water) was dropped onto the grid as a staining medium and the excess solution was removed with a filter paper. Samples were dried for 3 min, after which they were examined with the electron microscope and photographed at an accelerating voltage of 80 kV.

### 2.5. Determination of Encapsulation Efficiency and Loading Capacity

The amount of TQ or FITC encapsulated in coated or uncoated LP was determined by the dialysis bag method. The membrane (cut-off 3.5 kD) was filled with 2 mL of sample and kept for 1 h under magnetic stirring at 100 rpm and 25 °C, in 1L of deionized water, to remove free TQ. After that, the samples were diluted 1:50 with MeOH HPLC grade, sonicated for 30 min, and analyzed by HPLC-DAD. The EE% has been calculated as follows:

$$\mathrm{Encapsulation}\;\mathrm{efficiency}\%\;=\;\frac{\mathrm{TQ}\;\mathrm{encapulated}}{\mathrm{Total}\;\mathrm{TQ}}\;\times \;100$$

### 2.6. Storage Stability

LP-TQ and LP-TQ-HA storage stability studies were carried out for two months at +4 °C. Particle size, PdI, zeta-potential, and EE% were evaluated by DLS, ELS, and HPLC-DAD analyses. LP-FITC and LP-FITC-HA storage stability was monitored for 6 h.

### 2.7. In Vitro Release Study

Regenerated cellulose membranes with a molecular weight cut off 3.5–5 kD were selected to allow TQ diffusion into the acceptor medium, consisting of 200 mL PBS and Tween 80 0.5 % *w*/*v*. The dialysis bag was pre-hydrated for 30 min with deionized water, then filled with 2 mL of sample. The bag was doused into an acceptor medium, and the system was incubated at 37 °C under magnetic stirring for 6 h. At predetermined time intervals, 1 mL of the medium was withdrawn and replaced with the same volume of fresh release medium maintained at 37 °C to preserve sink conditions. TQ released at each time interval was quantified using HPLC-DAD.

### 2.8. Cell Culture Studies

#### 2.8.1. Human Corneal Epithelial Cells (HCE-2)

Human corneal epithelial cells (HCE-2) were cultured in serum-free medium supplemented with 0.05 mg/mL of BPE, 5 ng/mL of epidermal growth factor, 500 ng/mL of hydrocortisone, and 0.005 mg/mL of insulin. The cells were incubated at 37 °C in a humidified incubator containing 5% CO_2_ air and then plated on pre-treated 75 cm^2^ flasks with a mixture of 0.01 mg/mL of fibronectin, 0.03 mg/mL of bovine type I collagen, and 0.01 mg/mL of bovine serum albumin. The medium was changed twice a week. Once confluence was reached, the cells were split with a 1:3 ratio in other previously treated flasks.

#### 2.8.2. Human Conjunctival Epithelial Cells (HConEC)

Human conjunctival epithelial cells (HConEC) provided by Innoprot were isolated from the human conjunctiva. The cells were cultured in a culture medium consisting of the basal medium with the addition of the conjunctival epithelial cell growth supplement (CEpiCGS), penicillin/streptomycin (P/S), and fetal bovine serum, at 37 °C in a humidified incubator containing 5% CO_2_ air. The cells were plated in 75 cm^2^ flasks previously treated for 1 h with a 1:10 poly-lysine solution in PBS. The culture medium was changed twice a week. Once confluence was reached, the cells were split with a 1:3 ratio in other previously treated flasks.

#### 2.8.3. Analysis of In Vitro Cytotoxicity

Human corneal and conjunctival epithelial cells were suspended and plated in 24-well plates (approximately 4 × 10^4^ cells/cm^2^). Once they reached about 70–80% confluence, the medium was removed, and the cells were exposed to TQ, LP-TQ, and LP-TQ-HA suitably diluted in PBS. PBS was used as a positive control, and 0.01% BAK was used as a negative control for maximal toxicity. Corneal and conjunctival epithelial cells were incubated with TQ solution, TQ formulated in LP-TQ liposomes, or LP-TQ-HA hyaluronic acid-coated liposomes in different concentrations (10, 30 and 60 µM) and for two different periods of time (15 min and 1 h).

##### MTT Assay

The viability of conjunctival and corneal epithelial cells exposed for two different periods (15 min and 1 h) and in different concentrations of free or formulated TQ was evaluated by MTT assay. Cells were plated in 24-well plates and kept in culture. TQ, LP-TQ, and LP-TQ-HA were incubated in different concentrations for different periods in the vehicle. Part of the medium from each well was withdrawn and stored for the LDH assay. Cells were incubated with MTT at a concentration of 1 mg/mL [25]. After removing the MTT-containing solution, dimethyl sulfoxide (DMSO) was added to the wells to dissolve the formation, and the absorbance of MTT was read at 550 and 690 nm. The vehicle was used as a positive control. Cell viability was expressed as a percentage of cells incubated only in the vehicle at the corresponding exposure time.

##### LDH Assay

Damage in human corneal and conjunctival epithelial cells was quantitatively assessed by measuring the amount of LDH released by the damaged cells into the extracellular fluid, 15 min and 1 h after drug exposure, using the LDH kit, as previously described [26]. The LDH level corresponding to complete cell death was determined for each experiment by analyzing sister cultures exposed to 0.01% BAK. Background LDH release was determined in drug-unexposed control cultures and subtracted from all experimental values. The resulting values correlated linearly with the degree of cell loss estimated upon observation of cultures in phase contrast optics.

#### 2.8.4. Cellular Uptake Studies

For the evaluation of the intracellular content of FITC, the corneal epithelial cells (HCE-2) and the conjunctival cells (HConEC) were exposed for 1 h to LP-FITC or LP-FITC-HA containing 1 mg/mL of FITC, or to a saturated salution of fluorescent probe diluted in PBS. A qualitative assessment of FITC uptake at the cellular level was performed by culturing human corneal and conjunctival cells on histological slides, treated with free FITC or formulated on liposomes for 1 h and fixed in 4% formaldehyde in phosphate buffer 0.1 mol/L, pH 7.4, for 10 min; the cells were then stained with DAPI shielded fluorine (Sigma, Milan, Italy) to visualize the nucleus and were subsequently observed by fluorescence microscopy (Labophot-2 Nikon, Tokyo, Japan). Ten photomicrographs were randomly taken for each sample. Cell uptake was studied by fluorescence microscopy using FITC-labeled liposomes, with a high pressure mercury vapor lamp, 20× objective, NA = 0.75 (OLYMPUS BX3-CBH/U-MCZ). Filter set: excitation 365 nm emission 400 nm high pass DAPI, excitation 494 nm emission 518 nm.

## 3. Results and Discussion

### 3.1. Preparation and Characterization of TQ and FITC Liposomal Formulations

The first part of the study was aimed at selecting the constituents of the liposomal formulations. At first, phosphatidylcholine (PC) and cholesterol (CH) (4:1 weight ratio) were tested, and liposomes were obtained with good and homogenous dimensions and an EE% of 40% (Table 1 and Table 2). CH can decrease the loading properties of lipophilic drugs in liposomes, due to the rigidity it can impart to the bilayer [27]. Consequently, stearic acid, stearin, and linoleic acid, were used to improve the encapsulation efficacy and obtain stable drug delivery systems [28]. In this study Plurol Oleique or polyglycerolodioleate, HLB 3 [29], Maisine or glycerylmonolinoleate, HLB 1 [30], Transcutol P or diethylene glycol monoethyl ether, HLB 4 [31], Capryol 90, propylene glycol monocaprilat, HLB 5 [30], and Lauroglycol 90, glycopropylene monolaureate, HLB 3 [32] were selected to investigate the performance of the developed liposomes. The physical characteristics of 1 mg/mL TQ-loaded liposomes, prepared with the different liquid lipids, were compared to formulation containg PC plus CH.

#### 3.1.1. Physical Parameters of Liposomal Formulations

All formulations reported in Table 1 contained a gravimetric ratio of 4:1 PC:lipid. The liposomes containing CH had dimensions of 85 nm, while they ranged from 140 to 640 nm with the liquid lipids. The chemical composition of the various liquid lipids probably caused a change in the sizes of the bilayers by interacting with their lipophilic compartments. This also produced a worsening of the homogeneity of the sample, as evidenced by the PdI values. The formulations containing Capryol 90 and Lauroglycol 90 were discarded due to the sizes and the high PdI values, while the other formulations were considered and their encapsulation efficacy (EE%) compared.

#### 3.1.2. Chemical Characterization of Liposomal Formulations

All liposomes showed a good EE%, but the formulation with Plurol Oleique had greater EE% than the others. EE% was further increased with a 6:1 gravimetric ratio, reaching the value of 73%. Finally, the optimized liposomes contained 1 mg/mL of TQ, 600 mg of PC, and 100 mg of Plurol Oleique, doubling the TQ aqueous solubility. Table 3 reports the physical and chemical parameters of unloaded (LP) and TQ-loaded liposomes (TQ-LP).

#### 3.1.3. Characterization of Optimized Liposomal Formulations

Sizes and PdI values were optimized utilizing probe-type ultrasonication. Different sonication times ranging from 5 min to 2 min and 30 s were considered. it was noted that longer time sonication did not improve the liposomes’ physical parameters. The best experimental conditions were obtained with the 2 min and 30 s cycle, which included sonication intervals of 0.5 s alternating with 0.5 s rest periods. The liposomes have dimensions around 200 nm, and they are homogeneous.

The preparation of HA-coated liposomes (LP-TQ-HA) were obtained using the drop-by-drop coating method [24]. Different HA concentrations (0.1%, 0.2%, 0.5% *w*/*v*) were tested (Table 4). Selection criteria were based on physical parameters of liposomes (Table 4): HA 0.1% *w*/*v* produced liposomes with small sizes and a good PdI value. Higher concentrations led to an increase in size and above all a reduction in the homogeneity of the sample.

#### 3.1.4. Physical Parameters of HA-Coated Liposomes

Usually, HA coating is performed with covalent and non-covalent strategies. In this study, the LP-TQ had negative zeta-potential (Table 3), and HA was a negatively charged polymer; however, the HA coating is made possible by the interactions between PC and HA. PC competes for the hydrophobic centers along the HA chain, which are responsible for the inter- and intrachain interactions [33,34]. Hydrogen bonds have also been reported as the PC-HA interaction mechanism [35,36,37,38]. As shown in Table 3, after HA deposition, the particle sizes of liposomes increased from 146 ± 2 nm to 166 ± 3 nm. The zeta potential changed from −26 ± 0 to −36 ± 1 due to the anionic charge of HA. The high zeta-potential reduced the aggregation of the vesicles, increasing their stability, as also seen in stability studies.

Fluorescent liposomes (LP-FITC and LP-FITC-HA) were prepared to evaluate the uptake in Human Corneal Epithelial Cells (HCEC-2) and Human Conjunctival Epithelial Cells (HConEC). Their chemical and physical parameters are reported in Table 1. Both the formulations showed high EE%.

### 3.2. Morphology

**Figure 1:**
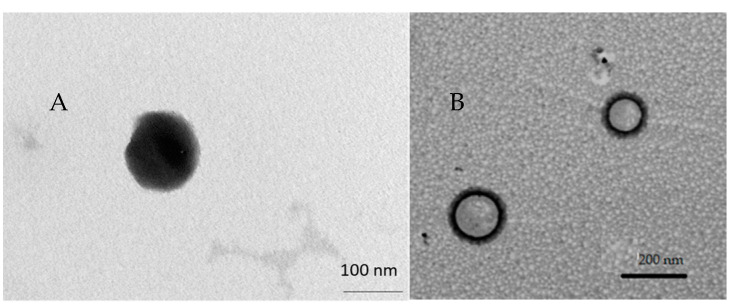
(**A**) Transmission electron microscope (TEM) image of TQ-loaded liposomes (LP-TQ); (**B**) TEM image of TQ-loaded liposomes coated with HA (LP-TQ-HA).

Transmission electron micrographs of LP-TQ showed vesicles with multi-layer structures (Figure 1) clearly distinct, with spherical shapes and nanosized dimensions, as suggested by DLS analysis. TEM of LP-TQ-HA showed a black a fluffy layer around the liposomes (Figure 1), not visible in LP-TQ, which confirms the deposition of the HA coating, in a similar manner to the results reported by several authors [39,40,41].

### 3.3. Stability Studies

Stability of the liposomal formulations was evaluated as a colloidal dispersion for 2 months in storage at +4 °C. The ability of the aqueous dispersions to maintain their physicochemical properties in terms of particle size, PdI, and drug entrapment was assessed. No significant changes were observed in the physical parameters of both LP-TQ and LP-TQ-HA dispersions (Table 5). In the case of LP-TQ, a decrease in EE% was observed, while the presence of the HA coating improved the chemical stability of the system, as compared to uncoated liposomes, maintaining the EE% close to 70% for 2 months. In the case of LP-TQ, the EE% after 2 months was about 50%.

#### Storage Stability Study of TQ Liposomes

For the in vitro uptake study on HCEC-2 and HConEC cells, the chemical-physical stability of LP-FITC and LP-FITC-HA over time was evaluated. Both liposomal formulations were stored at +4 °C for 6 h. Changes in size, PdI, or EE% were evaluated. The formulation maintained the initial characteristics, without the formation of any precipitate (Table 6).

### 3.4. In Vitro Release Study

**Figure 2:**
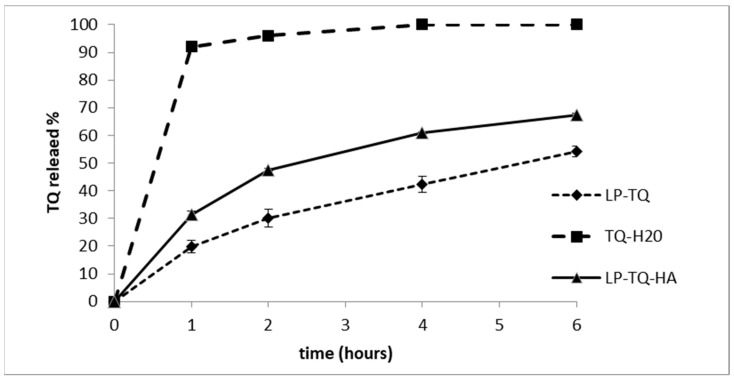
In vitro release profile of TQ from the LP-TQ, LP-TQ-HA, and TQ aqueous saturated solution in phosphate buffered saline (PBS) medium at pH 7.4, at 37 °C. Each value is the mean ± SD of three separate samples.

The dialysis bag method was employed to investigate the release of TQ from liposomes. The test was carried out in sink conditions and PBS (pH 7.4); 0.5% of Tween 80 was used as a release medium. The release profile of TQ from LP-TQ and LP-TQ-HA at 37 °C was compared with the release of a saturated aqueous solution (Figure 2).

The liposomes achieved a gradual and prolonged release of TQ over 6 h, while the release from the saturated solution occurred rapidly, with 100% of the TQ released after only 2 h. LP-TQ-HA were able to sustain the TQ release as well as non-coated liposomes with 67% and 54% of drug released over 6 h, respectively. This confirms that the addition of HA 0.1% *w*/*v* does not alter the release of TQ, as compared to the uncoated liposomes, and induces a more gradual release of the drug, compared to the saturated aqueous solution [39,42,43]. HA was observed to produce faster release than uncoated liposomes. This behavior has previously been reported in the literature [43].

In vitro release tests were also performed on the fluorescent formulations to evaluate the stability of FITC formulated in liposomes during the cellular experiments. The in vitro tests were carried out for 2 h at 37 °C. After 2 h, the percentage of FITC released from LP-FITC was 7%, while that from LP-FITC-HA was 8%, indicating that both systems, during the in vitro tests, were stable.

### 3.5. Cytotoxicity Studies in Human Epithelial Cells of Cornea (HCEC-2) and Conjunctiva (HConEC)

**Figure 3:**
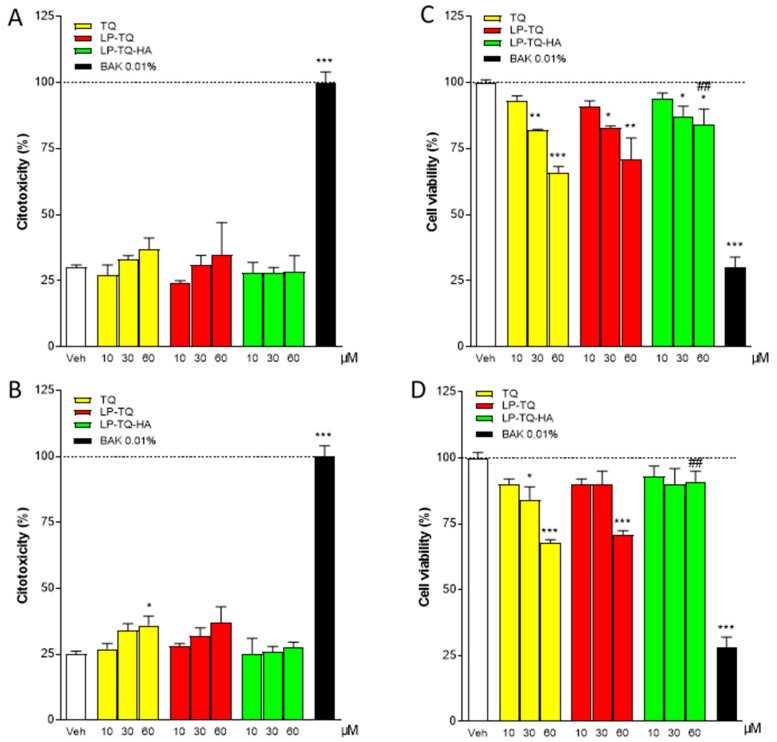
Evaluation of LDH and MTT assay in the human conjunctiva epithelial cells (HConEC) after 15 min (**A**,**C**) and 1 h (**B**,**D**) of incubation with TQ, LP-TQ, or LP-TQ-HA components. Data are expressed as percentages of the maximum degree of cell death BAK (**A**,**B**) and as percentages of the maximum cell viability (Veh) (**C**,**D**); they represent the mean ± standard error of the mean of at least 3 experiments performed in triplicate. * *p* < 0.05; ** *p* < 0.01, and *** *p* < 0.001 vs. Veh; ## *p* < 0.01 vs. 60 µM TQ free.

The cytotoxicity of TQ, both free and formulated in liposomes, was assessed with LDH and MTT assays, where 0.01% benzalkonium chloride (BAK) was considered the positive control. Figure 3 shows that at 15 min of incubation of the conjunctiva cells with free TQ or TQ-loaded liposomes, there are no changes in LDH levels (A), while 1 h of incubation of the same cells with 60 µM TQ induces a significant increase in LDH (B). The MTT assay of Figure 3 shows that free TQ already induces cellular distress at 15 min of incubation at concentrations of 30 and 60 µM; we observed similar effects with LP-TQ at the same concentrations (C). On the other hand, LP-TQ-HA does not show any toxicity in conjunctiva cells in any doses tested for 15 min and shows reduced toxicity at concentrations of 30 and 60 µM at the exposure time of one hour versus TQ (D).

**Figure 4:**
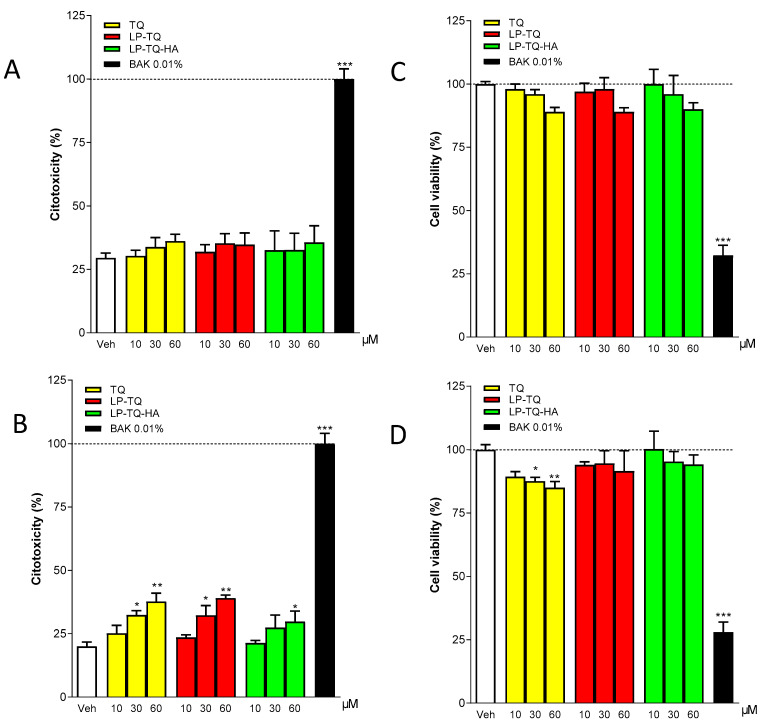
Evaluation of LDH and MTT assay in the human corneal epithelial cells (HCE-2) after 15 min (**A**,**C**) and 1 h (**B**,**D**) of incubation with TQ, LP-TQ, or LP-TQ-HA components. Data are expressed as percentages of the maximum degree of cell death BAK (**A**,**B**) and as percentages of the maximum cell viability (Veh) (**C**,**D**); they represent the mean ± standard error of the mean of at least 3 experiments performed in triplicate. * *p* < 0.05, ** *p* < 0.01, and *** *p* < 0.001 vs. Veh.

Corneal cells were more resistant to short time periods; we did not observe any toxicity for all the doses of the formulations tested for 15 min (Figure 4A,C). When the residence time was increased to 1 h, significant increases in LDH levels were observed for the free TQ and the TQ formulated in the liposomes at concentrations of 30 and 60 µM. The toxicity was significantly reduced with LP-TQ-HA, and only a slight increase in LDH was observed at concentrations of 60 µM (Figure 4B). The increased levels of LDH were observed in Figure 4B at high concentrations of the free TQ, and both the formulations resulted in altered levels of MTT only for TQ at the highest concentrations. The formulations reduced the TQ toxicity, which we observed at high doses and, in particular, in the case of LP-TQ-HA.

The in vitro release study (Figure 2) showed a different TQ release for LP-TQ and LP-TQ-HA at 24 h. MTT and LDH assays were performed for 15 min and 1 h. For these times, the quantity of TQ released by the two formulations was not very different. Both formulations guaranteed a more gradual release of the drug compared to the saturated aqueous solution, which released 90% of TQ at 1 h, and both formulations reduced the toxicity of TQ at high doses.

The data collected accord with the study by Hu X., which reports a slight toxicity of free TQ on human cells of the retinal pigment epithelium at 40 μM [6]. Our results show that HCEC-2 cells are more resistant to short exposure times than HConEC cells, since, in the former, no toxicity is observed for any of the tested formulations at any concentration used. Furthermore, the LP-TQ-HA is less toxic in both cell lines than the free TQ and the LP-TQ, as it is not toxic in the first 15 min in any of the doses tested, while when the residence time is increased to an hour, the toxicity increases slightly. Therefore, we can conclude that HA succeeds in reducing the toxicity of TQ at the level of HCEC 2 and HConEC cells. The reduction in cytotoxicity by HA was also evidenced by He M. et al. [44] in the case of nanoparticles. The liposome coating probably plays a key role in modulating the interactions between delivery system and cells, and hence in modifying the cytotoxicity of the formulation.

**Figure 5:**
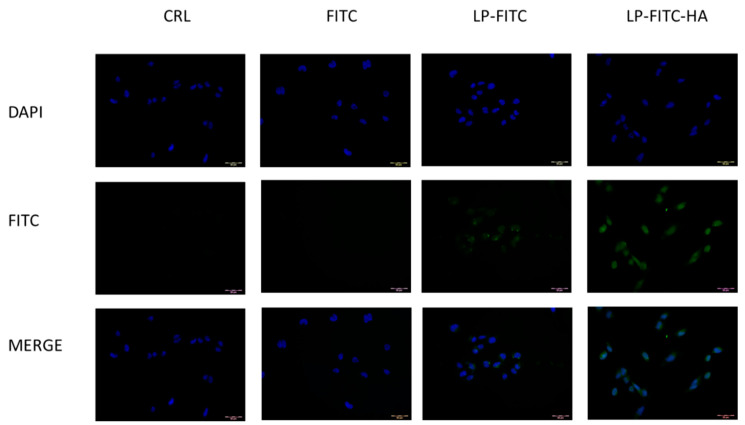
Cellular uptake of FITC, LP-FITC, and LP-FITC-HA by HConEC cells after 1 h incubation at 37 °C. Images of nuclei stained with DAPI (blue) and FITC (green) and their overlay. Scale bar: 20 μm.

**Figure 6:**
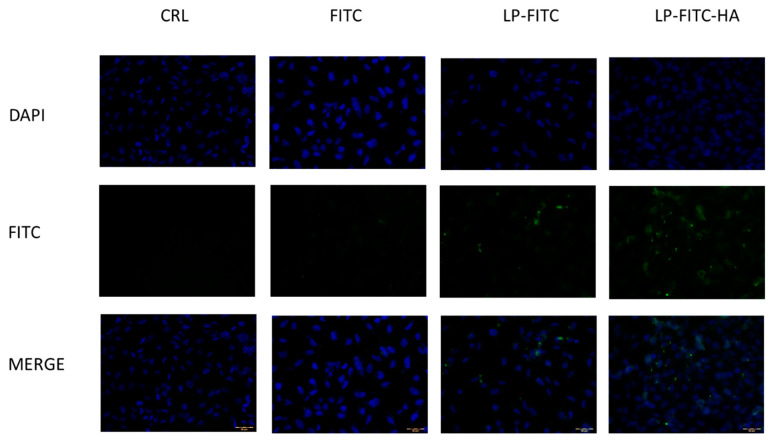
Cellular uptake of FITC, LP-FITC, and LP-FITC-HA by HCEC-2 cells after 1 h incubation at 37 °C. Images of nuclei stained with DAPI (blue) FITC (green) and their overlay. Scale bar: 20 μm.

From the previous tests, it can be seen that it is possible to reduce the cytotoxicity of TQ in HCEC-2 and HConEC cells. Up-take studies were carried out to understand if the formulations could improve the permeation of hydrophobic molecules such as TQ inside the cells. We prepared liposomes containing FITC, a fluorescent molecule with poor ability to enter cells, because of which the liposomes were coated with HA. Subsequently, we exposed HConEH cells to the free FITC, LP-FITC and LP-FITC-HA for one hour, the maximum time that we tested in the cytotoxicity assay. However, the photographs were taken under conditions consistent with unchanged detection parameters. The nucleus of each cell was labeled blue with DAPI. The free FITC cannot penetrate inside the cells, while when it is formulated in the liposomes, it manages to enter the cells (Figure 5). The intranuclear uptake of FITC loaded in liposomes was significantly greater than that of free dye at 1 h. Furthermore, the FITC formulated in LP-FITC-HA displayed stronger fluorescence intensity in the nucleus than LP-FITC. Similar results were obtained with corneal cells HCEC-2. The formulations increased the fluorescence inside the cells, which is evidence of the increased permeability of the FITC. The effect is more pronounced with HA-coated liposomes (Figure 6).

This finding is in agreement with the data reported in the literature, in which it was observed that HA-coated liposomes increase uptake at the cellular level; the increase is even more targeted at the nucleus level [43]. The increase in the uptake of LP-FITC-HA is due to the presence of HA receptors, CD44, and RHAMM on ophthalmic cellular tissues and, in particular, in human epithelial cells of the conjunctiva and cornea [19,20,45].

## 4. Conclusions

In this study, the potential of liposomes as a drug vehicle for the ophthalmic delivery of TQ was investigated. Two liposomal formulations have been designed and optimized. Both liposomes consist of phosphatidylcholine and Plurol Oleique, a liquid lipid which is used to replace cholesterol and ameliorate encapsulation efficiency and which doubles TQ solubility. This formulation was also coated with 0.1% *w*/*v* HA. Physical characterization revealed that uncoated and coated liposomes are suitable for ocular administration, with encapsulation efficiency of 70%. The formulations stored at +4 °C were stable for two months, and greater chemical stability was obtained with the HA coating. Liposomes guarantee a prolonged and gradual release, and they reduce the TQ toxicity observed at high dosage, particularly in the case of LP-TQ-HA when tested in both HCEC-2 and HConEC cells.

Finally, the in vitro uptake study, conducted with fluorescent liposomes, showed that both liposomal formulations increased the absorption at the cellular level and, in particular, at nucleus level, when tested in the corneal and conjunctival cells, with the most marked effect for HA-coated liposomes.

## Figures and Tables

**Table 1:** Physical characterization of different liposomal formulations (Mean ± SD, *n* = 3). PC: phosphatidylcholine, CH: Cholesterol.

Sample (4:1)	Size (nm)	PdI
PC:CH	85 ± 0	0.18 ± 0.01
PC:Plurol Oleique	141 ± 1	0.17 ± 0.02
PC:Maisine	232 ± 1	0.07 ± 0.01
PC:Trascutol P	182 ± 3	0.32 ± 0.03
PC:Capryol 90	516 ± 1	0.61 ± 0.01
PC:Lauroglycol 90	640 ± 7	0.64 ± 0.04

**Table 2:** Encapsulation efficiency (EE%) of different liposomal formulations (Mean ± SD, *n* = 3). PC: phosphatidylcholine, CH: Cholesterol.

Sample	EE%
PC:CH 4:1	40 ± 0
PC:Plurol Oleique 4:1	63 ± 5
PC:Plurol Oleique 6:1	73 ± 3
PC:Maisine 4:1	53 ± 6
PC:Trascutol P 4:1	40 ± 0

**Table 3:** Physical and chemical characterization of empty liposomes (LP), TQ- and fluorescein isothiocyanate (FITC)-loaded liposomes (LP-TQ and LP-FITC), and HA-coated liposomes (LP-TQ-HA and LP-FITC-HA) (Mean ± SD, *n* = 3).

Sample	Size (nm)	PdI	Zeta-Potential (mV)	EE%
LP	114 ± 3	0.23 ± 0.01	−23 ± 1	
LP-TQ	146 ± 2	0.15 ± 0.03	−26 ± 3	73 ± 3
LP-TQ-HA	166 ± 3	0.27 ± 0.01	−36 ± 1	73 ± 4
LP-FITC	106± 2	0.22 ± 0.01	−25 ± 2	98 ± 1
LP-FITC-HA	118 ± 1	0.25 ± 0.01	−34 ± 1	97 ± 2

**Table 4:** Physical characterization of LP-TQ-HA liposomes using different percentages of HA (Mean ± SD, *n* = 3).

HA % *w*/*v*	Size (nm)	PdI
0.1%	166 ± 3	0.27 ± 0.01
0.2%	558 ± 17	0.53 ± 0.07
0.5%	422 ± 23	0.49 ± 0.12

**Table 5:** Storage stability test of TQ-loaded liposomes (LP-TQ) and TQ-loaded liposomes coated with HA (LP-TQ-HA) at +4 °C over 2 months (Mean ± SD, *n* = 3).

Sample	Months	Size (nm)	PdI	EE%
LP-TQ	0	146 ± 2	0.15 ± 0.03	73 ± 3
	1	143 ± 2	0.18 ± 0.03	69 ± 2
	2	113 ± 3	0.23 ± 0.00	50 ± 1
LP-TQ-HA	0	166 ± 3	0.27 ± 0.01	73 ± 4
	1	160 ± 0	0.21 ± 0.01	71 ± 2
	2	156 ± 1	0.18 ± 0.01	68 ± 3

**Table 6:** Storage stability test of FITC-loaded liposomes (LP-FITC) and TQ-loaded liposomes coated with HA (LP-FITC-HA) at +4 °C for 6 days (Mean ± SD, *n* = 3).

Sample	Days	Size (nm)	PdI	EE%
LP-FITC	0	106 ± 2	0.22 ± 0.01	98 ± 1
	6	112 ± 1	0.23 ± 0.01	98 ± 0
LP-FITC-HA	0	118 ± 1	0.25 ± 0.01	98 ± 4
	6	125 ± 1	0.27 ± 0.01	97 ± 1

## Data Availability

The data presented in this study are available on request from the corresponding author.
